# The Role for Adjunctive Image in Pre-procedural Assessment and Peri-Procedural Management in Chronic Total Occlusion Recanalisation

**DOI:** 10.2174/1573403X10666140331143731

**Published:** 2014-05

**Authors:** Rodrigo Estevez-Loureiro, Matteo Ghione, Kadriye Kilickesmez, Pilar Agudo, Alistair Lindsay, Carlo Di Mario

**Affiliations:** NIHR Cardiovascular Biomedical Research Unit, Royal Brompton Hospital, London, United Kingdom

**Keywords:** CTO, MSCT, IVUS, PCI.

## Abstract

Non invasive coronary angiography with multislice computed tomography has exquisite sensitivity to detect calcium and even the faintest late contrast filling of the distal vessel. Calcium burden and occlusion length are still valuable markers of duration, complexity and success of the recanalisation procedure. The ability to visualise the vessel also in the occluded segment, especially if calcified, can also help the operator to understand where to pierce the proximal cap in stumpless occlusions and to predict unusual courses, especially in very tortuous arteries. Imaging side by side CT images and angiography during the recanalisation procedure is an established practice in many active CTO laboratories and algorithms for co-registration are designed to overcome the challenges of systo-diastolic and respiratory motion. Intravascular ultrasound is used in almost all cases by the experienced Japanese CTO operators but most of the times its main use is a better identification of the diseased segment after predilatation to ensure complete stent cover and appropriate stent expansion, an application similar to other complex non occlusive lesions. The specificity of IVUS during CTO recanalisation is the identification of the vessel path in stumpless occlusions and the guidance of wire reentry especially during reverse Controlled Retrograde Anterograde Tracking. Optical coherence tomography has limitations in the setting of CTO recanalisation because of the need of forceful contrast flushing to clear blood, contraindicated in the presence of anterograde dissections, and the limited penetration. The variability in the use of both non-invasive and invasive imaging during CTO recanalisation is immense, going from more than 90% in Japan to less than 20% in Europe and intermediate penetration in the USA. Probably the explanation is almost only in availability and cost because all countries see a progressive increase of use suggesting that these methods are becoming an established tool for guidance of CTO recanalisation.

## INTRODUCTION

In the recent years we have witnessed an enormous technological evolution in chronic total occlusion (CTO) dedicated material and techniques. These advances have permitted experienced operators to achieve successful recanalisation rates of >80% [1]. In particular novel dedicated guide wires have the steerability and power to be directed almost everywhere along the occluded segment but failure can still come when it is unclear where the occlusion starts (cap ambiguity), which direction to follow in long and tortuous occluded segments, how to orientate the wire to reenter in the true lumen, especially when poorly visualized angiographically. Intravascular imaging techniques can help each and any of these steps. It also serves a specific role in guiding balloon sizing and confirming the intravascular position of the anterograde wire in the technique which has become the gold standard to complete the procedure in retrograde CTO recanalisation, reverse Controlled Antegrade and Retrograde Tracking (CART). Adoption remains low because of the extreme complexity of the image interpretation but in expert hands may add a small fraction of percent of success and have the additional benefit to guide the final stent expansion, always difficult and potentially risky in very long segments with uneven diameter and possible negative remodeling. Guidance from non-invasive imaging is even more complex because no software has solved the monumental challenge to synchronise heart rate and respiratory excursion of previously recorded Multi-slice Computed Tomography (MSCT) and on-line angiography. Still information on localization and severity of calcification, true length of the occluded segment, path followed by the occlusion can be of help to plan the recanalisation procedures and understand the causes of failure of one strategy in order to overcome them or directly move to different approaches.

The aim of this paper is to review the role of these invasive and noninvasive imaging techniques in the percutaneous coronary treatment of CTO.

## NON-INVASIVE IMAGING: MULTI-SLICE COMPUTED TOMOGRAPHY (MSCT)

Non-invasive imaging is almost indispensable to define the indications to CTO, with cardiac magnetic resonance (CMR), stress echocardiography and nuclear perfusion scintigraphy challenging each other as the ideal technique to detect viability and ischaemia. CMR and echocardiography are also essential to detect complications of CTO recanalization, from pericardial effusion to septal haematomas. None of them, however, plays a role in the practical guidance of the recanalisation procedure with the possible exception of coronary MSCT.

**Fig. (1):**
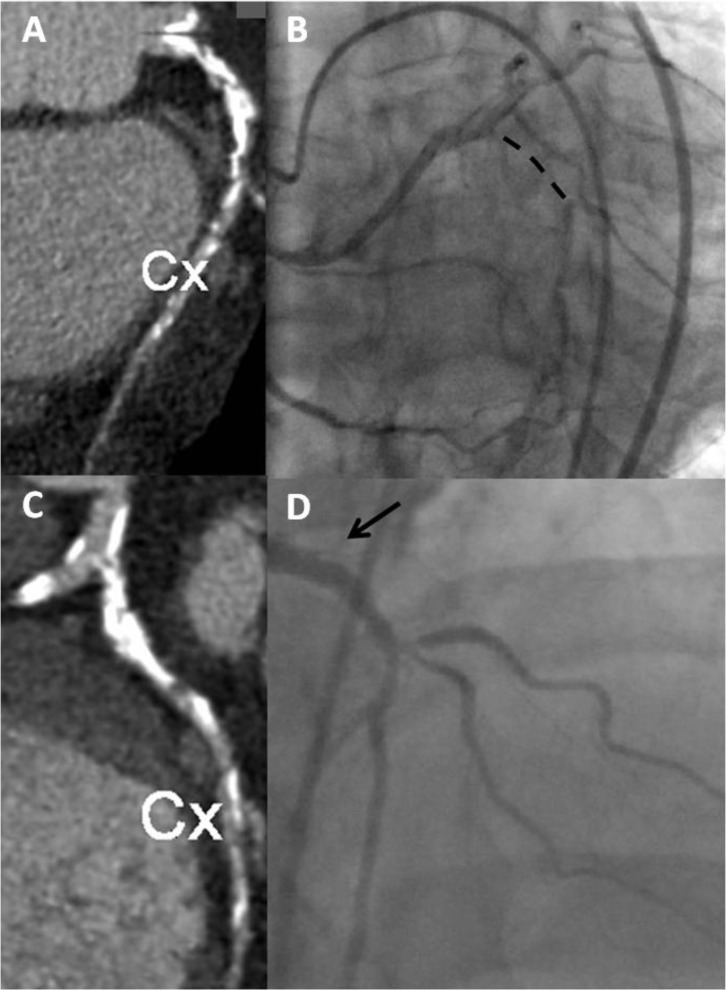
**Panel A. ** MSCT showing LAO caudal projection of the LCX artery. We observe a highly calcified lesion in the proximal LCX. **Panel B** shows a bilateral injection with the dashed line noting the length of the occlusion. **Panel C.** MSCT with LAO cranial orientation to depict the extreme calcification of the lesion. **Panel D** shows an angiographic LAO cranial projection with the arrow pointing to the stump of the occlusion. The appearance of the degree of calcification in the MSCT allows selecting the material to attempt the recanalisation and the expected rate of success.

MSCT angiography is an emerging reliable non-invasive imaging modality to detect presence, severity, lesion location of coronary artery stenoses [2-5]. Importantly, MSCT assesses not only luminal stenosis, but it also shows the plaque morphology in the vessel wall [6] and is more reliable than angiography to determine the total length of the obstruction. The appearance of a CTOs in MSCT is a complete interruption of contrast-enhanced lumen but it is not always possible to differentiate a truly CTO from a high degree coronary stenosis due to the limited spatial resolution of MSCT (Fig. **1**). True CTO lesions have been found to show more calcification, more marked reduction in contrast density and lesion length tends to be greater than 9 mm [7]. In the early stage of CTO PCI, several factors were associated with the possibility of failure in attempting a CTO recanalisation (such as moderate to severe calcium, occlusion length >20 mm, CTO stump not seen, missing or non-tapered, proximal tortuosity, antegrade bridging collaterals and presence of side branch at the occlusion site [8, 9]. PCI success decreases dramatically if one or more of these features are present [10]. However, with the current use of dedicated wires and microcatheters employed in modern recanalisation techniques the only factors independently associated with failure are the presence of severe calcification, the presence of a blunt stump and a length greater than 20mm when using an antegrade approach [10] and the tortuosity of collaterals when using a retrograde approach [11]. All of these characteristics, including the assessment of the largest epicardial collaterals, may be assessed by MSCT. MSCT is probably more useful in long, tortuous CTO lesions in which there is a need to characterize the path of the lesion and can accurately define the course of the occluded segment and allows better evaluation of the distal vessel. MSCT also allows the evaluation of the distribution of the calcium nodules inside the lesion, identifying features not readily seen by standard angiographic views [12]. It is not unusual that the true course and length of an occluded segment are very different with MSCT and selective coronary angiography. However, they are always apparent in MSCT and their visualization may be helpful for facilitating wire passage through the lesion [13]. The 3D nature of MSCT allows accurate measurements of length avoiding calibration limitations, poor collateral filling or lesion foreshortening, well known limitations of coronary angiography [14] MSCT can be as well used for providing guidance for the location of ostial occlusions, a feature difficulty seen in standard angiogram [13]. 

MSCT is more sensitive detecting, evaluating and localizing calcification in non-occluded vessels when compared to conventional angiography [14, 15]. MSCT has a great ability to characterize the plaque [16] composition (soft, mixed, fibro-calcified) and is the best available imaging modality to study the characteristics of the occluded segment.

All features described above are of great importance because their angiographic equivalents have been associated with worse outcome as previously described. This fact has lead to an increasing number of studies assessing the role of MSCT in characterizing CTO lesions and predicting success with percutaneous intervention. In the study of Mollet *et al*. [14] a total of 45 patients were evaluated before a CTO intervention. Results of this work showed that occlusion length >15mm and severe calcification defined as the presence of high-density plaques (≥130 HU) involving >50% of the coronary wall on a cross-sectional image and localized within the occlusion stump or occluded segment were independent predictors of PCI failure. Soon and coworkers [17] later studied 43 CTO lesions by means of 16-slice MSCT. They found that a transluminal calcification >50% was the only independent predictor of procedural failure, with a 10-fold increase in the probability of not crossing the lesion. Cho *et al.* [18] analised 64 patients with 72 CTO lesions by 64-slice MSCT with an overall procedural success of 76,3% . Almost all calcium parameters derived from the analysis were higher in patients with PCI failure. By multivariate regression analysis only the cross-sectional calcium area was independently associated with worse PCI outcome. Ehara *et al*. [19] performed an analysis of 110 patients with CTO lesions by using a 64-MSCT. The overall success rate of wire-crossing the lesion was 85%. They found that several MSCT-derived features were associated with an increased rate of failure. Bending, defined as > 45 degree either in the occluded site or proximal to the occlusion, shrinkage, defined as an abrupt narrowing or severe tapering of the distal portion with less than 1 mm in vessel diameter and severe calcification (defined as the presence of high-density (> 500 HU) plaques which nearly or completely occupy the vessel cross-section at the occluded site) were independently associated with the impossibility of the wire to cross the lesion. In this study, bending of the target vessel was the most powerful predictor of failure. Recently García-García reported the results of the CTTO registry [15], with regard to the use of MSCT in CTO recanalisation. Results from this registry show that occlusion length >15mm, severe calcification (defined as >50% of cross-sectional area) and presence of calcification at the entry point of the occlusion were associated with failure. Among them, the only independent predictor was the presence of severe calcification. The association between calcification at entry point and failure is of interest. MSCT may lead to an optimization in PCI strategy and to select the most suitable approach (antegrade/retrograde) by depicting the morphological features of the lesion so as to increase the probability of success. However, these strengths are counterbalanced by the fact that MSCT adds an additional dose of contrast and radiation to a procedure which is already intensive in both radiation and contrast dose. Until low dose acquisition can be reliably obtained, coronary MSCT should be done selectively and probably limited to patients that showed unfavorable features in standard angiography or in patients in whom a re-attempt is contemplated. 

MSCT undoubtedly offers some form of guidance during the procedure. By showing features such as calcification or tortuousity the operator can be induced to choose a different strategy, for instance using a retrograde approach to avoid a dense calcification at the entry point gaining reentry with reverse CART or using a knuckled wire to follow a tortuous path reducing the risk of perforation. During the procedure, dedicated coregistration software can display the MSCT images superimposed to the invasive angiography images and synchronized their rotation. This allows operators to better visualize the trajectory of the occluded segment, and to localize the calcified spots identified by MSCT in the invasive angiogram. This can be of particular interest in ostial occlusions or, in general, stumpless occlusions [20]. Unfortunately, no software so far has successfully overcome the challenge of synchronization of MSCT and angiographic images throughout the cardiac and the respiratory cycle. 

Ramcharitar [21] used MSCT overlay in combination with Stereotaxis, a system with magnets allowing three dimensional rotation of dedicated wires and showed that this technique is feasible in human CTOs with an overall success of 56%. However, there is still a need for high performance magnetic guidewire technology to achieve a success rate comparable to available sophisticated conventional CTO wires.

## INTRAVASCULAR ULTRASOUND (IVUS)

IVUS consists in of a miniaturized ultrasound transducer mounted on a transluminal catheter. It provides an axial spatial resolution of approximately 100-150 µm, adequate for direct imaging of the arterial wall [22]. Abrupt changes in acoustic impedance that occur at the interface between tissue types produce strong ultrasound reflection and generate an apparent border between anatomical compartments of the vessel wall. As it was stated before, the main cause of CTO recanalisation failure is the inability to pass the guidewire to the distal true lumen. Modern CTO operators have improved these results by using IVUS guidance to achieve this target [23-25]. There are three main applications of IVUS guided recanalisation of CTO. 

### Stumpless Occlusion

**Fig. (2):**
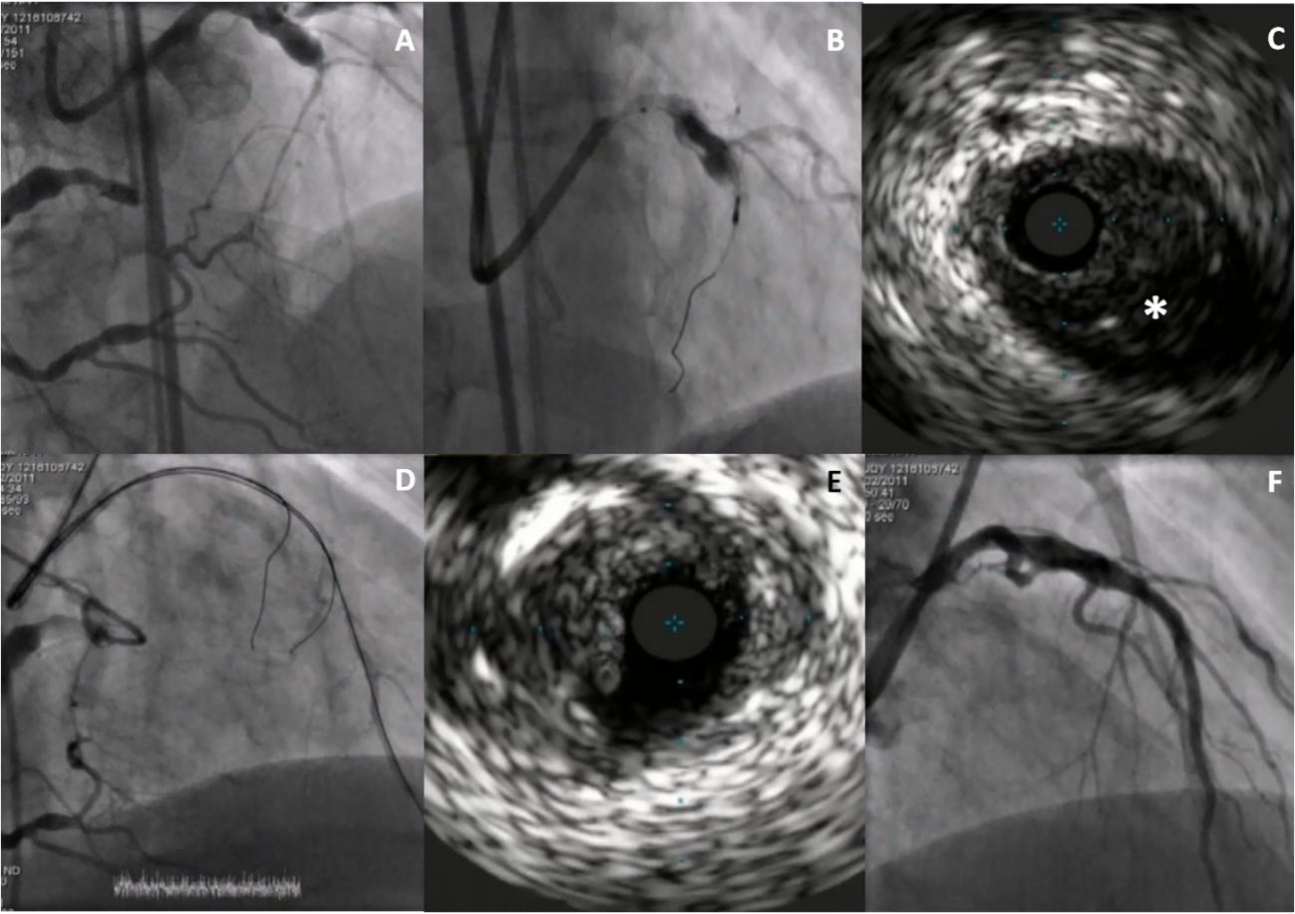
IVUS guided wiring of a stumpless CTO. **Panel A.** Bilateral injection showing the mid LAD occluded segment. **Panel B.** IVUS probe positioned in the septal branch at the point of entry of the occlusion. **Panel C.** IVUS imaging from the septal branch showing the entry point in LAD at 4 o´clock (asterisk). **Panel D.** Assisted by IVUS a wire could be placed in the true distal LAD. **Panel E.** IVUS imaging of LAD after predilatation with a 2.5 balloon. A small dissection can be noted. **Panel F.** Final angiographic result after LAD stenting.

Stumpless CTO are lesions when the localization of the entry point of the occlusion is not possible with angiography, despite the use of multiple angiographic views. If there is a sufficiently large branch originating at the point of occlusion site it is possible to advance the IVUS probe and identify the entry point during IVUS pullback. This may result in safer wire crossing and avoid complications such as perforations and severe coronary dissections. The IVUS transducer position can be roadmapped and the wire can be advanced in the direction of the entry point or the wire manipulation can be conducted under direct ultrasound guidance (Fig. **2**). In this last case, considering the size of a microcatheter required for wire manipulation, an 8 FR catheter is required. The morphological characteristics of the proximal cap might be helpful to choose the most appropriate wire. Once the wire penetrates the proximal entry, IVUS can be used to check the correct position of the wire in the true lumen [26]. This technique has been demonstrated to be associated with a recanalisation success of 81% in a recent series [27]. Fujii and coworkers analysed 67 native CTO lesions with IVUS after crossing the lesion with the guidewire or after dilation with a small balloon [28]. IVUS detected calcium in the occluded segment in 96% of cases (compared to 61% in non occlusive lesions) and was able to identify the proximal entry in all the cases. In the lesions with a side branch originating from the proximal end of the CTO, calcium was located more frequently opposite the side branch take-off. This may explain why guidewire penetration is more difficult in these situations since the wire would preferentially access the side branch. In 34% of penetration of the wire in the subintimal space during CTO recanalization was observed. In this series, inadequate flow after stenting was observed in 9% of lesions. And these lesions had longer stent length, longer distance between proximal and distal cap, larger distal plaque burden and larger total calcium index. However, the presence of hematoma was not associated with an impaired flow after stenting. 

### IVUS Guided Distal Reentry

The second main use of IVUS is to guide the wire positioning into the true lumen during an antegrade approach when a subintimal dissection has been created. When this occurs the IVUS probe can be advanced into the dissection and under direct ultrasound guidance a stiff wire can be manipulated to enter into the true lumen. To allow simultaneous insertion of a stiff, tapered wire over a microcatheter and the IVUS catheter, an 8 F guide is mandatory. A Volcano electronic transducer is preferred over mechanical catheters because of the shorter tip beyond the piezoelectric transducer. The IVUS catheter should be positioned at the re-entry point close to the distal cap, directing the wire opposite to the IVUS catheter toward the true lumen. Progression of the wire is then checked with IVUS advancing the IVUS probe in the occluded segment. Contrast injection should be withheld, especially if balloon dilation was required to advance the IVUS catheter [29]. One should be aware of potential complications, since sometimes false lumen dilation is required, this may increase the dissection and compromise the distal true lumen. A variant of this technique is applied when the wire enters a side branch in the occluded segment. The safest approach is to use a second wire and IVUS positioned in the side-branch. The complexity of this approach requires an expert CTO operator and an expert interpreter of the IVUS images working well coordinated. Calcium shielding the true lumen or the vessel and the second wire can complicate the procedure. This explains why other reentry methods such as the StingRay^®^ balloon and wire have been proposed and have substituted IVUS guidance in most centers.

### IVUS Guided Reverse CART

IVUS has been proposed to aid in the performance of a novel technique of retrograde CTO recanalisation [30]. Retrograde techniques have shown promising results using the retrograde collateral channels and, therefore, allowing the guidewire to reach the distal end of the CTO [11-31]. The CART technique consists of antegrade wiring through the CTO where retrograde balloon dilation creates a local subintimal dissection for facilitating wire crossing to the distal true lumen and was firstly described by Katoh and co-workers [32]. Since crossing fragile collateral channels with a balloon is difficult and risky, a dedicated microcatheter, called Corsair, has been developed and the preferred approach has become the opposite, retrograde reentry through the subintimal space created by a balloon deployed in the false lumen through an antegrade wire (reverse-CART). However, there are two potential drawbacks of crossing a CTO lesion with this technique. First, the subintimal space created can be too small if the size of the balloon selected is not big enough and second, the connecting subintimal channel may experience recoil with increasing difficulties to advance the retrograde wire. IVUS-guided reverse CART allows estimating the optimal size of the antegrade balloon matching vessel size based on the information on the true CTO vessel size, plaque components and distribution. This minimizes complications such as vessel perforation, excessive expansion of the subintimal dissection and contrast injection into the subintimal dissection. With this technique a 100% of success has been published in 31 cases of retrograde CTO crossing with low incidence of complications [30]. 

And finally, IVUS can be used to assess procedural complications that are sometimes negligible for standard angiography as some coronary perforations and intramural or extramural hematoma [33] and to optimize stent apposition and expansion, especially in complex cases of multiple stents or subintimal stenting [34]. However, in spite of these potential utilities, a recent consensus report does not recommend its use routinely in CTO PCI [34] and in the EuroCTO database 4-6% of the CTO procedures are guided by IVUS.

## OPTICAL COHERENCE TOMOGRAPHY (OCT)

**Fig. (3):**
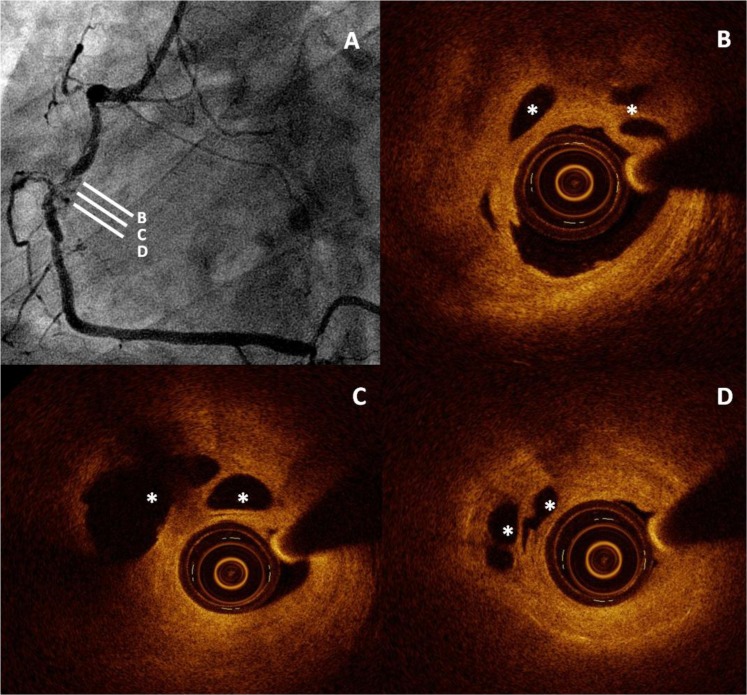
Optical Coherence Tomography (OCT) appearance of a CTO showing a significant amount of microchannels.** Panel A. **Angiographic apperance of the RCA occlusion.** Panels B, C, D**. OCT imaging showing a fibrous plaque with positive remodelling in the occluded segment and a significant amount of microchannels (asterisks). They are not in contact with the true lumen and there is no comunication between them. These features may help to distinguish them from dissections created after balloon dilatation.

OCT has become a key coronary imaging diagnostic tool and due to its greater spatial resolution (15 μm) is able to better characterize the processes of atherosclerotic plaques, providing additional detailed structural information over IVUS imaging [35, 36]**.** However, there is still limited information about its potential use during CTO recanalisation. There have been few case reports of the use of OCT by showing the position of the wire after crossing the lesion and predilation with regard to the true or false lumen [37], the result of stenting after subintimal tracking technique [38] or the histopathological correlates of CTO lesions by this technique (Fig. **3**) [39]. Nonetheless, there might be some concerns about the use of this intravascular imaging modality in this setting. This is due to the necessity of injecting large amount of contrast to obtain the images with a possible negative impact if some dissections have been created during the recanalisation. Future studies are warranted to clearly depict the role of this technique during CTP PCI.

## CONCLUSION

CTO is a complex scenario for PCI with lower success and higher rate of complications than in non-CTO vessels. However, percentages of failure have decreased over time with the new techniques of retrograde recanalisation and the new wires and microcatheters. Imaging analyses provide further characterization of the CTO lesion and may help to select those patients with more favorable anatomic features, to select those which can benefit more from the procedure, and to guide intervention in some circumstances (i.e. stumpless occlusion or wiring the true lumen form a subintimal dissection). Although should not be routinely used, they may be of invaluable utility in the most challenging cases.

## References

[1] Galassi AR, Tomasello SD, Reifart N  (2011). In-hospital outcomes of percutaneous coronary intervention in patients with chronic total occlusion: insights from the ERCTO (European Registry of Chronic Total Occlusion) registry.. EuroIntervention.

[2] Nieman K, Cademartiri F, Lemos PA, Raaijmakers R, Pattynama PM, de Feyter PJ (2002). Reliable noninvasive coronary angiography with fast submillimeter multislice spiral computed tomography.. Circulation.

[3] Ropers D, Baum U, Pohle K  (2003). Detection of coronary artery stenoses with thin-slice multi-detector row spiral computed tomography and multiplanar reconstruction.. Circulation.

[4] Kuettner A, Beck T, Drosch T  (2005). Diagnostic accuracy of noninvasive coronary imaging using 16-detector slice spiral computed tomography with 188 ms temporal resolution.. J Am Coll Cardiol.

[5] Miller JM, Rochitte CE, Dewey M  (2008). Diagnostic performance of coronary angiography by 64-row CT.. N Engl J Med.

[6] Schroeder S, Kuettner A, Leitritz M  (2004). Reliability of differentiating human coronary plaque morphology using contrast-enhanced multislice spiral computed tomography: a comparison with histology.. J Comput Assist Tomogr.

[7] Achenbach S, Daniel WG (2007). Current role of cardiac computed tomography.. Herz.

[8] Tan KH, Sulke N, Taub NA, Watts E, Karani S, Sowton E (1993). Determinants of success of coronary angioplasty in patients with a chronic total occlusion: a multiple logistic regression model to improve selection of patients.. Br Heart J.

[9] Puma JA, Sketch MHJr  (1995). Percutaneous revascularization of chronic coronary occlusions an overview.. J Am Coll Cardiol..

[10] Di Mario C, Werner GS, Sianos G  (2007). European perspective in the recanalisation of Chronic Total Occlusions (CTO) consensus document from the EuroCTO Club.. EuroIntervention.

[11] Rathore S, Katoh O, Matsuo H  (2009). Retrograde percutaneous recanalization of chronic total occlusion of the coronary arteries: procedural outcomes and predictors of success in contemporary practice.. Circ Cardiovasc Interv.

[12] Yokoyama N, Yamamoto Y, Suzuki S  (2006). Impact of 16-slice computed tomography in percutaneous coronary intervention of chronic total occlusions.. Catheter Cardiovasc Interv.

[13] Hecht HS (2008). Applications of multislice coronary computed tomographic angiography to percutaneous coronary intervention: how did we ever do without it?.. Catheter Cardiovasc Interv1.

[14] Mollet NR, Hoye A, Lemos PA  (2005). Value of preprocedure multislice computed tomographic coronary angiography to predict the outcome of percutaneous recanalization of chronic total occlusions.. Am J Cardiol.

[15] Garcia-Garcia HM, van Mieghem CA, Gonzalo N  (2009). Computed tomography in total coronary occlusions (CTTO registry): radiation exposure and predictors of successful percutaneous intervention.. EuroIntervention.

[16] Pundziute G, Schuijf JD, Jukema JW  (2008). Head-to-head comparison of coronary plaque evaluation between multislice computed tomography and intravascular ultrasound radiofrequency data analysis.. JACC Cardiovasc Interv.

[17] Soon KH, Cox N, Wong A  (2007). CT coronary angiography predicts the outcome of percutaneous coronary intervention of chronic total occlusion.. J Interv Cardiol.

[18] Cho JR, Kim YJ, Ahn CM  (2010). Quantification of regional calcium burden in chronic total occlusion by 64-slice multi-detector computed tomography and procedural outcomes of percutaneous coronary intervention.. Int J Cardiol.

[19] Ehara M, Terashima M, Kawai M  (2009). Impact of multislice computed tomography to estimate difficulty in wire crossing in percutaneous coronary intervention for chronic total occlusion.. J Invasive Cardiol.

[20] Magro M, Schultz C, Simsek C  (2010). Computed tomography as a tool for percutaneous coronary intervention of chronic total occlusions.. EuroIntervention.

[21] Ramcharitar S, van der Giessen WJ, van der Ent M, de Feyter P, Serruys PW, van Geuns RJ (2011). The feasibility and safety of applying the Magnetic Navigation System to manage chronically occluded vessels: a single centre experience.. EuroIntervention.

[22] Yock PG, Fitzgerald PJ (1998). Intravascular ultrasound: state of the art and future directions.. Am J Cardiol.

[23] Ito S, Suzuki T, Ito T  (2004). Novel technique using intravascular ultrasound-guided guidewire cross in coronary intervention for uncrossable chronic total occlusions.. Circ J.

[24] Matsubara T, Murata A, Kanyama H, Ogino A (2004). IVUS-guided wiring technique: promising approach for the chronic total occlusion.. Catheter Cardiovasc Interv.

[25] Furuichi S, Airoldi F, Colombo A (2007). Intravascular ultrasound-guided wiring for chronic total occlusion.. Catheter Cardiovasc Interv.

[26] Rathore S, Terashima M, Suzuki T (2009). Value of intravascular ultrasound in the management of coronary chronic total occlusions.. Catheter Cardiovasc Interv.

[27] Park Y, Park HS, Jang GL  (2011). Intravascular ultrasound guided recanalization of stumpless chronic total occlusion.. Int J Cardiol.

[28] Fujii K, Ochiai M, Mintz GS  (2006). Procedural implications of intravascular ultrasound morphologic features of chronic total coronary occlusions.. Am J Cardiol.

[29] Yamane M (2012). Current percutaneous recanalization of coronary chronic total occlusion.. Rev Esp Cardiol.

[30] Rathore S, Katoh O, Tuschikane E, Oida A, Suzuki T, Takase S (2010). A novel modification of the retrograde approach for the recanalization of chronic total occlusion of the coronary arteries intravascular ultrasound-guided reverse controlled antegrade and retrograde tracking.. JACC Cardiovasc Interv.

[31] Sianos G, Barlis P, Di Mario C  (2008). European experience with the retrograde approach for the recanalisation of coronary artery chronic total occlusions.. A report on behalf of the euro CTO club. EuroIntervention.

[32] Surmely JF, Tsuchikane E, Katoh O  (2006). New concept for CTO recanalization using controlled antegrade and retrograde subintimal tracking: the CART technique.. J Invasive Cardiol.

[33] Tsujita K, Maehara A, Mintz GS  (2009). Intravascular ultrasound comparison of the retrograde versus antegrade approach to percutaneous intervention for chronic total coronary occlusions.. JACC Cardiovasc Interv.

[34] Sianos G, Werner GS, Galassi AR  (2012). Recanalisation of chronic total coronary occlusions: 2012 consensus document from the Euro CTO club.. EuroIntervention.

[35] Jang IK, Bouma BE, Kang DH  (2002). Visualization of coronary atherosclerotic plaques in patients using optical coherence tomography: comparison with intravascular ultrasound.. J Am Coll Cardiol.

[36] Prati F, Guagliumi G, Mintz GS  (2012). Expert review document part 2: methodology. terminology and clinical applications of optical coherence tomography for the assessment of interventional procedures.. Eur Heart J.

[37] Schultz C, van der Ent M, Serruys PW, Regar E (2009). Optical coherence tomography to guide treatment of chronic occlusions?.. JACC Cardiovasc Interv.

[38] Niccoli G, Ferrante G, Galassi AR, Montone RA, Crea F (2010). Optical coherence tomography follow-up of the subintimal tracking and re-entry technique for chronic total occlusion.. EuroIntervention.

[39] Teijeiro Mestre R, Alegria-Barrero E, Di Mario C (2013). Microchannels in Recent Chronic Total Occlusions Assessed With Frequency-Domain Optical Coherence Tomography.. Rev Esp Cardiol.

